# Modification of *Brassica rapa* L. Polysaccharide by Selenylation and Its Immune-Enhancing Activity When Combined with a Live-Attenuated Newcastle Disease Vaccine in Poultry

**DOI:** 10.3390/ani15182755

**Published:** 2025-09-21

**Authors:** Sijia Wang, Jungang Wang, Hong Shen

**Affiliations:** Collage of Animal Science & Technology, Shihezi University, Shihezi 832003, China; sijiawang0922@163.com

**Keywords:** selenium modification, *Brassica rapa* L. polysaccharide, lymphocyte proliferation, oral vaccine immunoenhancer

## Abstract

**Background**: The quest for effective immunoenhancers is central to improving vaccine efficacy, especially against avian viruses such as Newcastle disease (ND) virus. Selenized polysaccharides integrate bioactive polysaccharides with selenium’s immunoenhancing properties while reducing selenium toxicity, making them promising candidates for the development of a novel vaccine immunoenhancer. **Aim**: This study aimed to develop an efficient selenized *Brassica rapa* L. polysaccharide (sBRP) and evaluate its potential to enhance the immunogenicity of a live-attenuated ND vaccine in poultry. **Methods**: Selenization was achieved via nitrite-assisted selenization of *Brassica rapa* L. polysaccharide (BRP). In vivo, 180 yellow-feathered broilers were divided into six groups: control (Con), vaccine-only (Vac), BRP (20 mg/kg), and low/medium/high-dose sBRP (sBRP-L/M/H: 5/10/20 mg/kg). On days 14 and 28, all groups except Con were vaccinated against ND via drinking water. Concurrently, the BRP and sBRP-L/M/H groups received their respective polysaccharides via oral gavage. Parameters assessed included immune organ indices, lymphocyte proliferation, serum antibody titers (HI), cytokine levels (IL-2/IL-6/IFN-γ), and densities of intestinal intraepithelial lymphocytes (IELs) and goblet cells (GCs). **Results**: sBRP exhibited a selenium content of 30.6 mg/g, with Se-O-C covalent modification confirmed. The sBRP-H group significantly enhanced immune organ indices, lymphocyte proliferation, Newcastle disease virus HI antibody titers, and serum IL-2/IL-6/IFN-γ levels. The sBRP-M group increased IEL and GC densities in the intestine. **Conclusions**: sBRP acts synergistically with the vaccine to enhance vaccine-induced cellular, humoral, and mucosal immunity, demonstrating promise as a novel oral vaccine immunoenhancer.

## Simple Summary

Newcastle disease is a highly contagious virus that poses a major threat to the poultry industry worldwide. Vaccination is the primary control measure; however, vaccines administered through drinking water often suffer from weak immunogenicity and provide short-lived protection, highlighting the need for effective immunoenhancers to elicit a stronger and more durable immune response. To address this limitation, a novel immunoenhancer was developed through the selenization of a natural *Brassica rapa* L. polysaccharide, yielding selenized polysaccharide (sBRP). This study aimed to evaluate the potential of sBRP to enhance the immune response of a live-attenuated Newcastle disease vaccine following oral co-administration in broilers. The results demonstrated that broilers receiving the vaccine co-administered with sBRP exhibited significantly elevated antibody titers compared to those immunized with the vaccine alone. Furthermore, sBRP supplementation markedly improved gut mucosal immunity, a crucial frontline defense mechanism against enteric pathogens. These findings demonstrate that sBRP is a safe and effective oral immunoenhancer, providing a scientific basis for its application to optimize vaccination strategies in the poultry industry.

## 1. Introduction

Immunosuppressive diseases are widely recognized for their severity, not only severely hindering the development of the livestock industry but also causing significant losses to businesses [1,2,3]. Currently, due to the lack of specific preventive and therapeutic methods, the control of such diseases largely relies on vaccines [3,4]. However, while most vaccines exhibit high antigen specificity, their immunogenicity is generally low, often requiring the use of immunostimulants to achieve optimal immune responses [5,6,7]. Although chemical immunostimulants such as aluminum adjuvants, Freund’s adjuvants, and oil adjuvants are widely used in clinical settings, their inherent tendency for bioaccumulation, limited target spectrum, and adverse effects restrict their long-term application prospects [8]. Against this backdrop, exploring natural drug resources to obtain safe, efficient, and multi-targeted immunoenhancers has become an important research direction [9,10]. Multiple studies have revealed that the active components in traditional Chinese herbs possess powerful immune-modulating functions, capable of synergistically enhancing both specific immunity (e.g., promoting T/B cell responses) and non-specific immunity (e.g., activating macrophage and NK cell functions), while significantly improving immune organ indices and cytokine release levels [11,12,13].

*Brassica rapa* L. polysaccharide (BRP), also known as turnip or rutabaga, belongs to the Brassicaceae family and the Brassica genus. It is a commonly used medicinal and edible plant among residents of Xinjiang, China, with both its seeds and roots being used for medicinal purposes. The active compounds in *Brassica rapa* L. include polysaccharides, flavonoids, and glucosinolates. Numerous studies have demonstrated that BRP, as biological macromolecules, not only provide energy to the body but also possess physiological functions such as antioxidant, antitumor, immune regulation, and hypoglycemic effects, making them highly valuable for application [14,15]. Additionally, studies demonstrate that BRP engages receptors expressed on immune cell membranes [16]. This interaction stimulates various immune cells, including macrophages, natural killer cells, T lymphocytes, and B lymphocytes. Consequently, this activation cascade leads to a potentiation of the organism’s immunological defenses [17].

As an indispensable trace element in numerous biological systems, selenium functions as an integral constituent of vital antioxidant enzymes including glutathione peroxidase and thioredoxin reductase, and plays a role in regulating immune responses, antioxidant activity, and inhibiting cell apoptosis [18]. Selenium exists in both inorganic and organic forms. However, due to the accumulation and toxicity of inorganic selenium, its bioavailability is low. In contrast, organic selenium forms have lower toxicity and can more efficiently exert beneficial biological activities, such as selenomethionine (SeMet), selenocysteine (SeCys), and selenopolysaccharides [19]. Among these, selenium-modified polysaccharides not only significantly enhance safety but also combine the biological functions of both polysaccharides and selenium [20,21]. Especially in key biological functions such as immune regulation and antioxidant activity, selenium-modified polysaccharides demonstrate significant advantages. The enhancing effect of selenium modification on polysaccharide immune activity has been supported by experimental evidence. For example, Liu and colleagues found that feeding chickens with selenium-enriched Lin’s layer fungus selenium polysaccharides significantly improved immune parameters and antioxidant status in laying hens [22]; similarly, Gao and others experimentally confirmed that selenium-modified Codonopsis pilosula polysaccharides exhibit significantly enhanced immune-modulating activity [23]. Therefore, selenium polysaccharides emerge as an ideal candidate for developing an efficient and safe immunopotentiator.

Accordingly, the present study was conducted to develop and characterize a sBRP and to assess its efficacy as an immunoenhancer for the live-attenuated Newcastle disease vaccine in poultry.

## 2. Materials and Methods

### 2.1. Preparation of BRP

Based on the previous report [24], *Brassica rapa* L. was extracted using an optimized ultrasonic-assisted extraction (UAE) procedure and purified by the trichloroacetic acid (TCA) method to remove proteins [25]. The extraction was carried out at a solid-to-liquid ratio of 20:1 (mL/g), an extraction temperature of 60 °C, and an ultrasonic power of 160 W for 40 min. Deproteinization was achieved through two sequential applications of the TCA method, followed by collection of the resulting supernatant. This supernatant was then dialyzed using a regenerated cellulose dialysis bag (14 kDa) with milli-Q water (18.2 MΩ·cm at 25 °C) at 4 °C for 48 h, with the ultra-pure water replaced every 6 h. The resulting polysaccharide solution is concentrated by rotary evaporation and freeze-dried under vacuum to obtain BRP, which is weighed and stored for later use.

### 2.2. Preparation of sBRP

This experiment employed the nitric acid-sodium selenite method [26] (HNO_3_-Na_2_SeO_3_) for selenisation modification of BRP. Three levels of three factors were selected for selenisation: sodium selenite (0.05 g/mL) addition amount (A): 4 mL, 6 mL, 8 mL; reaction temperature (B): 40 °C, 60 °C, 80 °C; and reaction time (C): 4 h, 6 h, 8 h, to conduct an L_9_ (3^4^) orthogonal experiment. After mixing the polysaccharide solution with sodium selenite and reacting, the pH was adjusted to 5–6 using Na_2_CO_3_. Subsequent centrifugation at 1000× *g* for 10 min yielded the supernatant, which was then placed in a regenerated cellulose dialysis bag (14 kD) for continuous dialysis. The presence of SeO_3_^2−^ in the dialysate was detected using the ascorbic acid method [27], with 6 h per cycle, until the solution no longer showed red color (no residual SeO_3_^2−^), at which point dialysis was stopped. The dialysate was concentrated under reduced pressure using a rotary evaporator and subsequently lyophilized in a freeze dryer (BTP-8ZLOVX-1 Freeze Dryer, SP Scientific, Warminster, PA, USA). This process yielded nine selenized BRP (sBRP) fractions, which were designated sBRP_1_–sBRP_9_.

### 2.3. Determination of Carbohydrate and Selenium Content in sBRP

Carbohydrate concentration in serial samples (sBRP_1_–sBRP_9_) was quantified employing the phenol-sulphuric acid colorimetric assay [28]. Standard glucose solutions of different concentrations were prepared, phenol and concentrated sulphuric acid were added, and the absorbance was measured at 490 nm. A standard curve for glucose was then plotted. The linear regression equation was y = 13.979x + 0.1498 (R^2^ = 0.9989). The absorbance of each sample was measured using the same procedure, and the carbohydrate content of sBRP_1_–sBRP_9_ was calculated based on the standard curve.

The selenium content of sBRP_1_–sBRP_9_ was determined using the hydride generation–atomic fluorescence spectroscopy method. Using selenium standard solutions, a standard curve was plotted with selenium mass concentration as the x-axis and fluorescence intensity as the y-axis, and the regression equation was established: y = 55.052x + 10.903 (R^2^ = 0.9969). The fluorescence intensity of each sample was measured using the same procedure, and the selenium content was calculated by substituting the values into the equation. Finally, the sample with the highest selenium content was selected from sBRP_1_ to sBRP_9_ and named sBRP, and further characterization was performed.

### 2.4. sBRP Characterization

#### 2.4.1. Fourier Transform Infrared Spectroscopy (FT-IR)

Fourier transform infrared (FTIR) spectroscopy was performed on the BRP and sBRP samples using the KBr pellet method. Briefly, 2 mg of each sample was thoroughly ground with dry potassium bromide (KBr) in an agate mortar and subsequently pressed into a transparent pellet. FTIR spectra were then acquired using a spectrometer over a wavenumber range of 4000 to 400 cm^−1^ (BRUKER Fourier transform Raman spectrometer, Bruker Optics GmbH, Ettlingen, Germany).

#### 2.4.2. Field Emission Electron Scanning Electron Microscope (SEM)

The surface morphology of the BRP and sBRP samples was scanned by field emission scanning electron microscopy (SU8010 FESEM, Hitachi Limited, Tokyo, Japan). Prior to observation, the samples were coated with a thin layer of gold via ion sputtering. The observations were conducted at an acceleration voltage of 5 kV.

#### 2.4.3. Zeta Potential and Particle Size

BRP and sBRP were prepared as aqueous solutions (2.0 mg/mL) and centrifuged prior to analysis. The particle size and zeta potential of the samples were then measured at 25 °C using a dynamic light scattering analyzer (Zetasizer Ultra, Malvern Panalytical, Malvern, Worcestershire, UK).

#### 2.4.4. Solid-State Nuclear Magnetic Resonance (NMR)

sBRP was dissolved in heavy water (D_2_O) and added to a nuclear magnetic resonance tube. Its ^1^H-NMR and ^13^C-NMR were measured using a spectrometer (Spinsolve 80 Carbon Desktop NMR Spectrometer, Magritek, Aachen, Germany).

### 2.5. Animals and Experimental Design

A total of 180 one-day-old, unvaccinated, healthy, male, fast-growing yellow-feathered broilers were sourced from a commercial poultry farm in Beiquan Town, Shihezi City. The chicks were housed in triple-tiered iron cages (2 m × 0.9 m × 1.8 m) under controlled environmental conditions. The ambient temperature was maintained at 32–35 °C on the first day and gradually reduced to a stable temperature of 22 °C by the final two weeks of the trial. All chicks had ad libitum access to feed and water. Strict biosecurity and disinfection protocols were implemented throughout the study period.

The chicks were fed until 14 days of age (maternal antibodies at 2.5 log2), after which the 180 chicks were randomly allocated into six groups, six replicates per group. Except for the blank control group, the remaining groups were vaccinated via drinking water on the 14th and 28th days, respectively. (Newcastle Disease Vaccine, Live, Batch No. 202049, Strain La Sota, Shandong Huahong Bio-engineering Co., Ltd., Binzhou, China). All groups received their respective treatments via oral gavage once daily for five consecutive days, starting concurrently with each vaccination: the polysaccharide groups received 0.5 mL of BRP (20 mg/kg) or sBRP (5, 10, or 20 mg/kg), while the vaccine control group (Vac) and blank control groups (Con) received an equal volume of physiological saline (the doses were selected based on the results of a pilot study). The whole experiment process continued for 42 days. The experimental groups and administration procedures are detailed in Table 1.

#### 2.5.1. Effects of sBRP on Chicken Body Weight and Immune Organ Indices

The body weight of each group of broiler chickens was measured at the time of the first vaccination. On days 14 (D_14_) and 28 (D_28_) after the first vaccination, following euthanasia, six chickens were randomly selected from each group, weighed, and their thymus, spleen, and bursa of Fabricius were removed and weighed to calculate the immune organ indices. Especially, the entire thymus (all lobes) was collected from each chicken, and adherent adipose and connective tissues were meticulously removed by gentle scraping with sterile scalpels and forceps. The cleaned thymus was then blotted on sterile filter paper to remove any residual fluid and weighed immediately to obtain the wet weight for index calculation.

The immune organ index = immune organ weight (mg)/body weight (g).

#### 2.5.2. Peripheral Lymphocyte Proliferation Assay (A_570_)

Starting from the day of the first immunization (designated as Day _0_, which corresponded to day 14 of age), blood samples (5 mL) were collected aseptically via cardiac puncture from six randomly selected chickens per group at 7, 14, 21, and 28 days post-initial immunization (D_7_, D_14_, D_21_, D_28_). These time points corresponded to broiler ages of 21, 28, 35, and 42 days, respectively. The peripheral lymphocyte proliferation assay was performed using the CCK-8 method. The absorbance (A_570_) at 570 nm was measured using an enzyme-linked immunosorbent assay (ELISA) reader, and the lymphocyte proliferation rate was calculated. (Multiskan FC Microplate Reader, Thermo Fisher Scientific, Waltham, MA, USA).

$$\mathrm{Lymphocyte}\;\mathrm{proliferation}\;\mathrm{rate}\;(\%)=({\bar{A}}_{polysaccharide}-{\bar{A}}_{control})/{\bar{A}}_{control}\times 100\%$$

#### 2.5.3. Serum HI Antibody Titre Testing

Seven (D_7_), 14 (D_14_), 21 (D_21_), and 28 (D_28_) days after the first vaccination, six chickens were randomly selected from each group for blood sample collection. The blood samples were allowed to clot at room temperature for 2 h and then centrifuged at 1000× *g* for 10 min to obtain the serum. And the serum hemagglutination inhibition antibody titer was detected using a microtiter assay [29].

#### 2.5.4. Serum Cytokine Content Determination

On days 14 (D_14_) and 28 (D_28_) after the first vaccination, six chickens were randomly selected from each group, blood samples were collected and centrifuged (Heraeus Megafuge 8R, Thermo Fisher Scientific, Osterode am Harz, Germany) to collect serum and the serum IL-2, IL-6, and IFN-γ contents were determined using enzyme-linked immunosorbent assay (ELISA) (Shanghai Enzyme-linked Biotechnology, Shanghai, China).

#### 2.5.5. Density of Intestinal Mucosal Intraepithelial Lymphocytes (IELs) and Goblet Cells (GCs)

At D_14_ and D_28_, five chickens per group were randomly selected. A ~5 cm jejunal segment was dissected and fixed in 4% paraformaldehyde. Within 48 h, tissue sections were prepared using hematoxylin-eosin (HE) staining for IELs and periodic acid-Schiff (PAS) staining for GCs. Whole-slide imaging was performed using a digital slide scanner for comprehensive histological acquisition (PANNORAMIC DESK/MIDI/250/1000, 3DHISTECH Ltd., Budapest, Hungary). The number of lymphocytes/ goblet cells and corresponding epithelial length in each section were quantified uniformly in millimeters (mm) using Image-Pro Plus 6.0 software. Densities were calculated as follows:

IELs Density (cells/mm) = Number of Lymphocytes (cells)/Epithelial Length (mm)

GCs Density (cells/mm) = Number of Goblet Cells (cells)/Epithelial Length (mm)

### 2.6. Statistical Analysis

Statistical analysis was performed using SPSS software (version 27.0). Data are presented as the mean ± SEM. The normality of data distribution for all datasets was confirmed by the Shapiro–Wilk test (*p* > 0.05). The homogeneity of variances was assessed using Levene’s test. For datasets where the assumption of homogeneity was met (*p* > 0.05), differences among groups were analyzed by one-way ANOVA followed by Tukey’s HSD post hoc test. For datasets where this assumption was violated (*p* < 0.05), the Welch ANOVA was performed followed by the Games–Howell post hoc test. A *p*-value < 0.05 was considered statistically significant.

## 3. Results

### 3.1. Yield, Sugar Content, and Selenium Content of sBRP

As shown in Table 2, sBRP_1_ had the highest yield at 36.66%, followed by sBRP_4_ and sBRP_9_. sBRP_1_ also had the highest sugar content at 65%, followed by sBRP_2_, sBRP_3_, and sBRP_5_. sBRP_5_ had the highest selenium content at 30.6 mg/g, followed by sBRP_1_, sBRP_6_, and sBRP_3_.

Taking selenium content as the primary factor, analysis of the K-values (Table 2) from the orthogonal experiments indicated that the factors influencing sBRP selenium content can be determined as: reaction time > sodium selenite addition amount > reaction temperature. And theoretical optimal conditions for maximizing selenium content were a reaction time of 8 h, a sodium selenite addition amount of 6 mL, and a reaction temperature of 40 °C.

Importantly, studies have shown that the immunostimulatory activity of selenized polysaccharides is positively correlated with their selenium content, with the highest selenium derivatives demonstrating the most potent effects [30]. Therefore, we used sBRP_5_ for the following study.

### 3.2. sBRP Characterization Results and Analysis

#### 3.2.1. FT-IR Analysis

**Figure 1:**
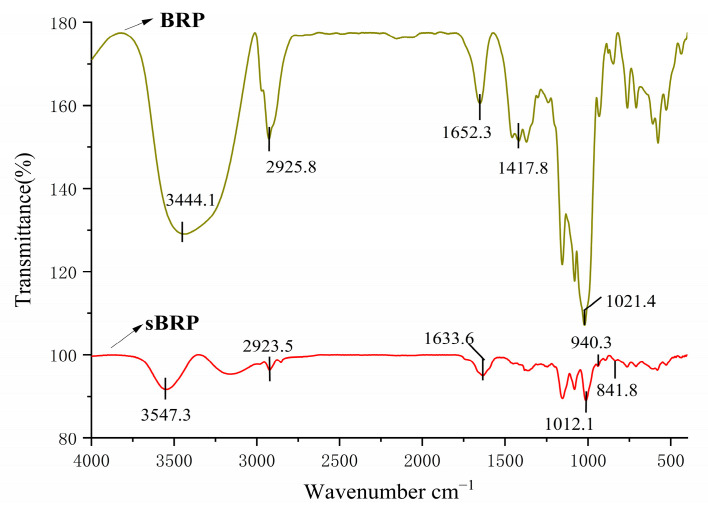
Infrared spectra of BRP and sBRP.

In the infrared spectrum shown in Figure 1, the characteristic peaks at 3444.1 cm^−1^ (BRP) and 3547.3 cm^−1^ (sBRP) correspond to the characteristic absorption peaks of -OH stretching vibrations (3600–3200 cm^−1^). Additionally, both BRP and sBRP exhibit strong peaks in the C-O-C bond range of 1400–100 cm^−1^, indicating that both BRP and sBRP are polysaccharides. BRP and SBRP both exhibit absorption peaks near 2925 cm^−1^ (BRP: 2925.8 cm^−1^, sBRP: 2923.5 cm^−1^), corresponding to the C-H stretching vibrations of methylene and methyl (-CH_3_) groups. However, the peak positions show minimal changes after selenation, indicating that the carbon skeleton of the polysaccharide main chain has not undergone significant destruction. After selenisation, the C-O-C peak of the polysaccharide shifts to 1012.1 cm^−1^, a lower wavenumber compared to before selenisation (1021.4 cm^−1^), suggesting that selenium may bind to the polysaccharide in the form of selenite esters. Additionally, sBRP exhibits characteristic absorption peaks of the polysaccharide, along with new weak absorption peaks at 940.3 cm^−1^ and 841.8 cm^−1^, which align with the vibration modes of the Se-O-C and Se-O characteristic peaks, indicating that the polysaccharide has been successfully selenated.

#### 3.2.2. Scanning Electron Microscopy (SEM) Image Analysis

**Figure 2:**
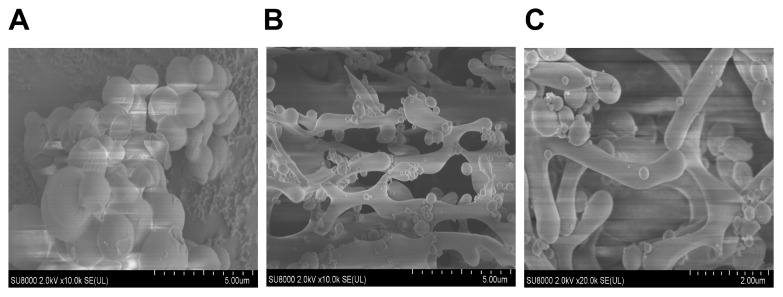
(**A**) Scanning electron microscopic image of BRP at 10,000 magnification. (**B**) Scanning electron microscopic image of sBRP at 10,000 magnification. (**C**) Scanning electron microscopic image of sBRP at 20,000 magnification.

Figure 2A shows that BRP exists as uniform spherical particles with a smooth surface and tightly connected structures, featuring small pores and no obvious particles. In Figure 2B, selenium-treated sBRP exhibits a loose network structure with irregularly sized spherical protrusions on the surface, resulting in significantly increased surface roughness and enlarged pores. Upon further magnification, Figure 2C reveals that sBRP also exhibits a layered stacking structure. The cross-linking and layered structure of sBRP may be due to selenium treatment altering the original intermolecular hydrogen bonds or electrostatic interactions of the BRP, or it may be attributed to the hydrophobic interactions formed by the binding of selenium to the polysaccharide.

#### 3.2.3. Zeta Potential and Particle Size Analysis

**Figure 3:**
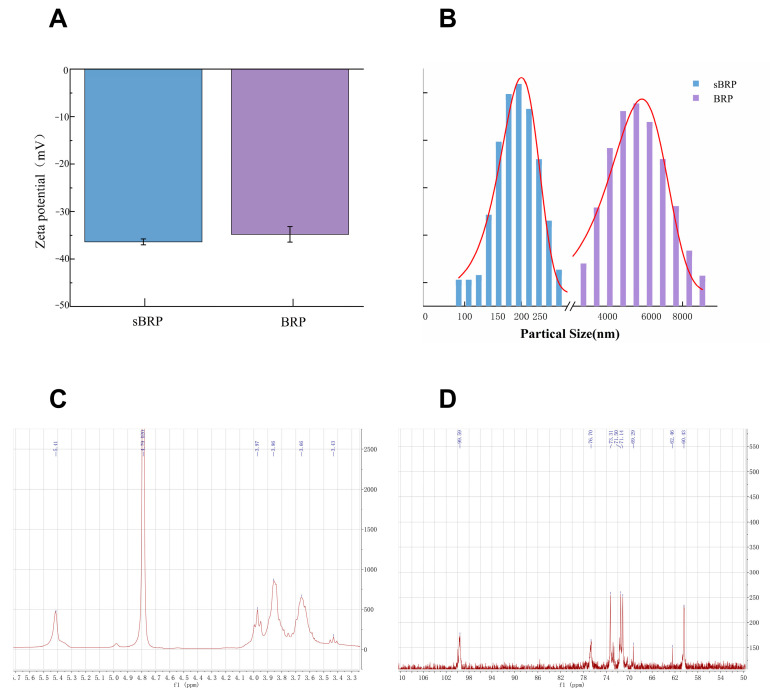
(**A**) Zeta potential map of BRP and sBRP. Data represent the mean±SEM of three technical replicate measurements from a single representative sample. (**B**) Particle size distribution chart of BRP and sBRP. (**C**) The ^1^H NMR spectra of sBRP. (**D**) The ^13^C NMR spectra of sBRP.

As shown in Figure 3A, at a concentration of 2 mg/mL, the average Zeta potential of BRP was −34.8 ± 1.64 mV, while that of sBRP was −36.4 ± 0.62 mV. The absolute value of the Zeta potential of sBRP was 11.64% higher than that of BRP. As shown in Figure 3B, the average particle size of BRP was 5077.64 ± 114.97 nm, while that of sBRP was 200.04 ± 3.28 nm, significantly smaller than that of selenium-free polysaccharides. These results indicate that the introduction of selenium in BRP increases their surface negative charge density, enhances colloidal stability, and alters their higher-order structure, making them more likely to form tighter aggregates with smaller particle sizes.

#### 3.2.4. NMR Analysis

As shown in Figure 3C, the chemical shifts in the ^1^H-NMR of sBRP are 3.43, 3.66, 3.86, 3.97, and 5.41 ppm, all of which indicate that this substance is a polysaccharide. Among these, the chemical shifts at 3.43, 3.66, 3.86, and 3.97 ppm originate from the H2–H6 protons on the polysaccharide sugar ring. Generally, the chemical shift in the terminal hydrogen in the 4.9–4.0 ppm range corresponds to the β configuration, while the 5.5–5.0 ppm range corresponds to the α configuration. The terminal hydrogen chemical shift in sBRP is 5.41 ppm, indicating an α-glycosidic bond.

As shown in Figure 3D, the chemical shifts in the ^13^C-NMR spectrum of sBRP are 60.43, 62.46, 69.29, 71.14, 71.50, 73.31, 76.70, and 99.59 ppm. The C-6 signal splits into 60.43 and 62.46 ppm, indicating that part of the C-6 hydroxyl group is replaced by a selenate group, and the electron-withdrawing effect causes the signal to shift to a lower field. The chemical shifts in the anomeric carbon in polysaccharides generally range from 90 to 110 ppm, while the signals of non-anomeric carbon range from 60 to 85 ppm. Among these, the signals of the anomeric carbon in the α configuration appear between 95 and 103 ppm, while those in the β configuration appear above 103 ppm. The signal at 99.59 ppm corresponds to the C-1 position of the anomeric carbon, and this is further confirmed by the hydrogen spectrum, verifying that sBRP adopts the α configuration.

### 3.3. The Effect of sBRP on Broiler Weight and Immune Organ Indices

**Figure 4:**
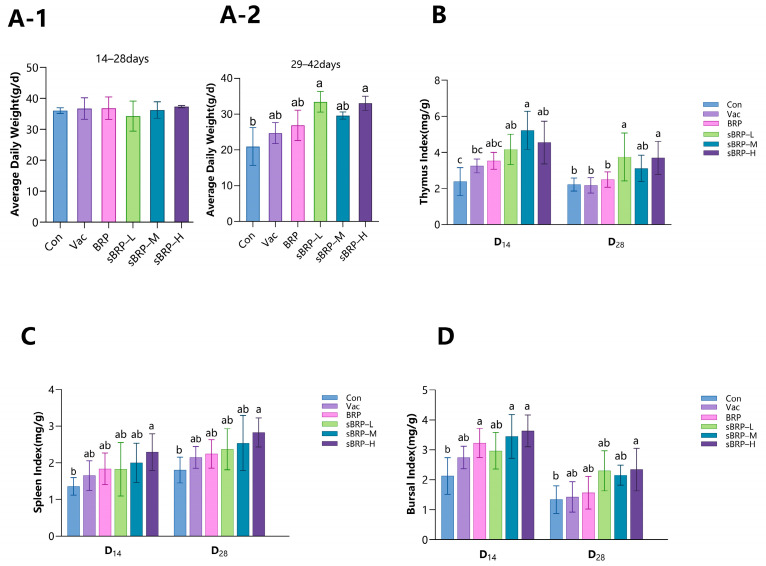
(**A-1**) ADG of 14–28 days; (**A-2**) ADG of 29–42 days. (**B**) Thymus index. (**C**) Spleen index. (**D**) Bursal index. Data are presented as mean ± SEM (*n* = 6). Bars in same time point without the same superscripts (a–c) differ significantly (*p* < 0.05, ANOVA).

During the initial growth phase (days 14–28), no statistically significant variations in average daily gain (ADG) were observed across experimental groups (Figure 4A-1). Subsequently (days 29–42), chickens administered sBRP exhibited enhanced growth rates compared to both Con and Vac groups. Specifically, sBRP-L and sBRP-H groups demonstrated significantly greater mass increments relative to Con groups (*p* < 0.05).

At 14 days after the first sBRP administration (D_14_), sBRP-treated groups exhibited significantly elevated thymus indices relative to Con groups (*p* < 0.05). Furthermore, the sBRP-M group demonstrated substantially increased thymic mass measurements compared to the Vac group (*p* < 0.05); at 14 days after the second sBRP administration (D_28_), the sBRP-L and sBRP-H groups maintained significantly higher thymic indices than Con, Vac, and BRP groups (*p* < 0.05) (Figure 4B). While the sBRP-M group showed numerically greater thymus indices than the Con, Vac and BRP groups at this stage, these differences did not achieve statistical significance (*p* > 0.05).

The sBRP-H group maintained peak splenic indices throughout the experiment, with values that were statistically significantly elevated compared to the Con group at both 14 days after the first and second administration of sBRP (*p* < 0.05) (Figure 4C). Moreover, all sBRP-treated groups exhibited higher splenic indices after the booster administration compared to their levels post-primary administration.

At 14 days after the first sBRP administration (D_14_), the sBRP-H group had the highest bursa of Fabricius index, followed by the sBRP-M and BRP groups, all of which were significantly higher than the Con group; at 14 days after the second sBRP administration (D_28_), the sBRP-H group had significantly higher bursa of Fabricius indices than the Con group (*p* < 0.05). (Figure 4D).

### 3.4. Changes in Peripheral Lymphocyte Proliferation

Changes in lymphocyte A_570_ values for each group are shown in Table 3. From D_14_ to D_28_, the lymphocyte A_570_ values in the sBRP-H group were significantly higher than those in the Con group (*p* < 0.05). The lymphocyte A_570_ values did not differ significantly between the selenized polysaccharide and BRP groups from D_7_ to D_28_. However, the values in the selenized polysaccharide groups were numerically higher at all measured time points.

**Figure 5:**
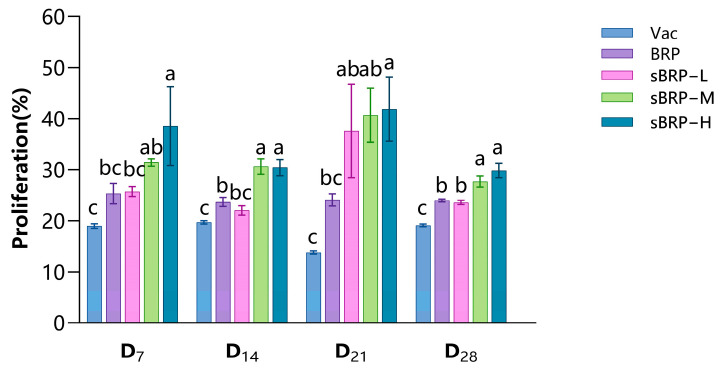
Lymphocyte proliferation rate of every group. Data are presented as mean ± SEM (*n* = 6). Bars at the same time point without the same superscripts (a–c) differ significantly (*p* < 0.05, ANOVA).

The lymphocyte proliferation rates in the experimental chickens of each group are shown in Figure 5. The sBRP-H group exhibited the highest lymphocyte proliferation rates throughout the entire experimental period, which were significantly higher than those of the Con, Vac, and BRP groups (*p* < 0.05). The sBRP-M group showed higher lymphocyte proliferation rates than the Con group from D_7_ to D_28_, and significantly higher than the Vac group at D_14_ and D_28_ (*p* < 0.05).

### 3.5. Changes in Serum Antibody Titres

Serum antibody titers for all groups are detailed in Table 4. The sBRP groups consistently elicited significantly elevated antibody responses relative to the Con group across all dosage levels and temporal measurements. Specifically, the sBRP-H group demonstrated markedly higher titers than both Con and Vac groups at every post-vaccination interval (*p* < 0.05). Peak immunoreactivity occurred at day 21, with sBRP-H titers significantly surpassing BRP values at D_14_, D_21_, and D_28_ (*p* < 0.05).

### 3.6. Serum Cytokine Levels

#### 3.6.1. Changes in Serum IL-2 Levels

**Figure 6:**
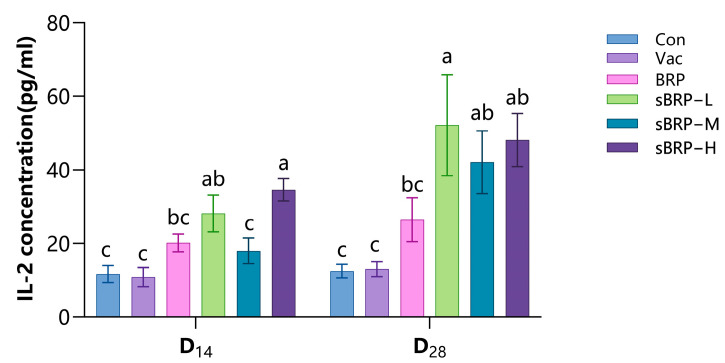
The serum IL-2 contents of every group. Data are presented as mean ± SEM (*n* = 6). Bars at the same time point without the same superscripts (a–c) differ significantly (*p* < 0.05, ANOVA).

Serum IL-2 concentrations across groups are presented in Figure 6. At D_14_, both sBRP-L and sBRP-H groups demonstrated significantly elevated cytokine concentrations relative to Con and Vac groups. The sBRP-H group additionally exhibited higher IL-2 levels than the BRP group (*p* < 0.05); at D_28_, all sBRP-treated groups showed enhanced IL-2 production compared to both Con and Vac groups, with sBRP-L values significantly surpassing BRP measurements (*p* < 0.05).

#### 3.6.2. Changes in Serum IL-6 Levels

**Figure 7:**
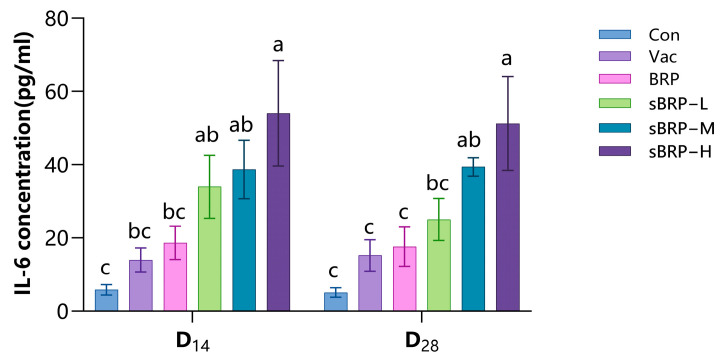
The serum IL-6 contents of every group. Data are presented as mean ± SEM (*n* = 6). Bars at the same time point without the same superscripts (a–c) differ significantly (*p* < 0.05, ANOVA).

Serum IL-6 levels in each group are shown in Figure 7. At each measurement interval, all sBRP-treated groups demonstrated significantly elevated serum IL-6 concentrations relative to control animals. Furthermore, the high-dosage sBRP group (sBRP-H) exhibited substantially increased cytokine levels compared to both Vac and BRP groups (*p* < 0.05).

#### 3.6.3. Changes in Serum IFN-γ Levels

**Figure 8:**
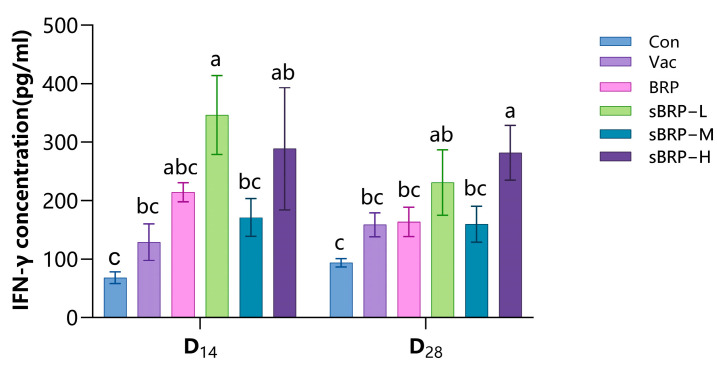
The serum IFN-γ contents of every group. Data are presented as mean ± SEM (*n* = 6). Bars at the same time point without the same superscripts (a–c) differ significantly (*p* < 0.05, ANOVA).

Serum IFN-γ levels in each group are shown in Figure 8. Both sBRP-L and sBRP-H groups exhibited significantly elevated cytokine concentrations compared to controls at each measurement interval (*p* < 0.05); at D_28_, the sBRP-H group demonstrated robust increases in IFN-γ levels relative to Con, Vac, and BRP groups.

### 3.7. The Changes in the Density of Intraepithelial Lymphocytes (IELs) and Goblet Cells (GCs) in the Jejunal Tissue of Chickens

**Figure 9:**
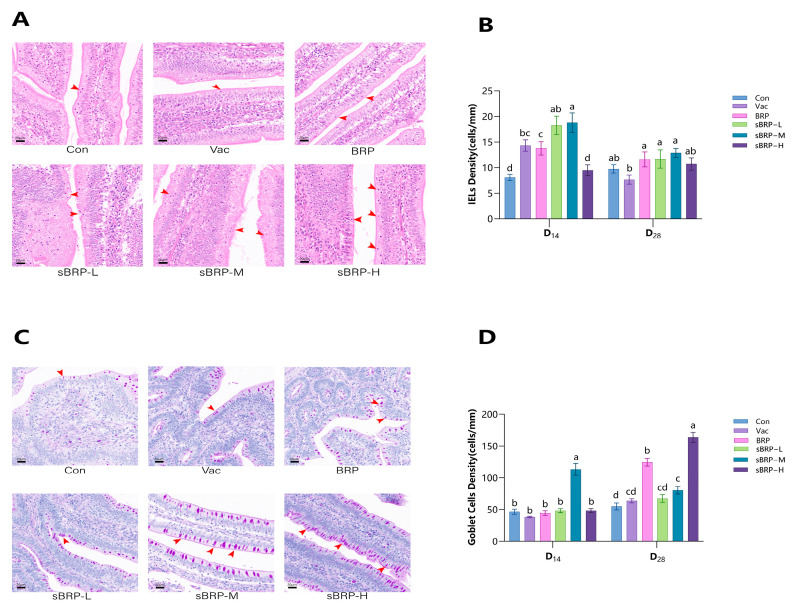
(**A**) Representative micrograph showing HE IELs (red arrows) within the epithelial cell layer in the jejunum. Scale bars: 20 μm. (**B**) Quantitative analysis of IELs density. (**C**) Representative micrograph showing PAS goblet cells (red arrows indicate PAS-positive goblet cells) within jejunum. Scale bars: 20 μm. (**D**) Quantitative analysis of goblet cell density. (**B**,**D**) Data are presented as mean ± SEM (*n* = 5). Bars at the same time point without the same superscripts (a–d) differ significantly (*p* < 0.05, ANOVA).

As shown in Figure 9B, at D_14_, the IEL densities in both the sBRP-L and sBRP-M groups were significantly higher than those in the BRP group (*p* < 0.05), while the densities in the BRP, sBRP-L, and sBRP-M groups were all markedly elevated compared to the Con group (*p* < 0.05). By D_28_, the overall IEL density had decreased, although the densities in the sBRP-L and sBRP-M groups remained significantly greater than that in the Vac group (*p* < 0.05). Additionally, at D_14_, the GC density in the sBRP-M group was significantly greater than that in the Con, Vac, and BRP groups. At D_28_, compared to the Vac group, both the BRP and sBRP-H groups exhibited enhanced mucosal immune defense, as evidenced by a significant increase in GCs density (*p* < 0.05). Furthermore, the GC density in the sBRP-H group was significantly higher than that in the BRP group (*p* < 0.05).

## 4. Discussion

Natural polysaccharides are often limited in their applications due to drawbacks such as high molecular weight and poor solubility. However, chemical modification can introduce polar groups to improve water solubility and bioavailability [31,32]. Selenium is a core component of glutathione peroxidase and possesses strong antioxidant capabilities. Selenium-modified polysaccharides can combine the immune-modulating properties of polysaccharides with the antioxidant functions of selenium, thereby creating a ‘dual-activity synergistic effect [33]. Currently, common methods for selenium modification include the SeOcl_2_ method and the HNO_3_-NaSeO_3_ method [34]. Due to its low cost, mild reaction conditions, and clear reaction sites, the HNO_3_-NaSeO_3_ method is convenient for parameter optimization through orthogonal experiments. Liu conducted orthogonal experiments on the selenisation of Atractylodes macrocephala polysaccharides, selecting three factors: sodium selenite dosage, reaction time, and reaction temperature. The optimal conditions for the highest selenium content (12.23 mg/g) were determined to be a sodium selenite dosage of 200 mg, a reaction time of 6 h, and a reaction temperature of 70 °C [35]. Therefore, in this experiment, three factors with significant influence on the reaction—sodium selenite dosage, reaction temperature, and reaction time—were selected for selenisation modification of BRP. Using selenium content as the indicator, the influence factors were determined through range analysis: Time > (0.05 g/mL) Na_2_SeO_3_ > Temperature. The optimal reaction conditions were a reaction time of 8 h, Na_2_SeO_3_ (0.05 g/mL) addition amount of 6 mL, and a reaction temperature of 40 °C. The highest selenium content of the selenium polysaccharides prepared in this experiment reached 30.6 mg/g. Studies have shown that the selenium content of selenium polysaccharides depends on the source of the natural polysaccharides and the selenisation method [33]. For example, under the same conditions, the selenium conjugation capacity of Chinese angelica polysaccharides (6990–12,330 μg/g) is more than 10 times higher than that of Codonopsis pilosula polysaccharides (478 μg/g) [36,37].

The structure-activity relationship of polysaccharides is primarily determined by their structural heterogeneity, which also accounts for their diverse biological activities [38,39]. Comprehensive analysis using SEM, Zeta potential and particle size, FT-IR, and NMR revealed that selenium modification introduces selenate groups into the polysaccharide backbone via substitution of the C-6 hydroxyl group, forming a porous network structure (Figure 2). This structural change enhances antioxidant capacity by increasing the exposure of active sites. Additionally, the Zeta potential decreased from −34.8 to −36.4 mV (Figure 3), indicating an increase in negative charge density. This was further confirmed by the Se-O-C characteristic peak (940.3 cm^−1^) in FTIR and the shift in the C-6 signal (62.46 ppm) in NMR, both of which validate the successful introduction of the selenate group. Selenium modification significantly reduced the particle size of BRP, with smaller particle sizes generally considered to be more easily absorbed by the body and to possess stronger pharmacological activity [40].

Selenium, as an essential trace element for animal organisms, maintains homeostasis by participating in various biochemical reactions. However, its toxicity exhibits chemical form dependency [41]. Research indicates that the methylation rate and excretion efficiency of selenium jointly determine its biosafety [42,43]. When the intake exceeds 3 mg/kg BW, it exhibits teratogenic effects on poultry [44]. In this experiment, the effective dose of selenium in the sBRP-H group with the highest concentration was 0.612 mg/kg, which is below the teratogenic dose. Additionally, the experimental results showed that during the 29–42-day period, the ADG of all sBRP groups was higher than that of the control group. Therefore, it can be concluded that sBRP, as a potential oral immune stimulator and enhancer of the Newcastle vaccine, has no adverse effects on broiler chickens and has the potential to promote animal growth.

Immune organ indices are pivotal biomarkers for avian immunological status. Elevated thymic index signifies enhanced T-cell maturation [45]; increased splenic index reflects activated humoral/cellular immune responses [46]; and higher bursal index directly correlates with B-cell differentiation efficiency [47]—collectively indicating strengthened host defense capabilities. The thymus and spleen indices in the selenium-polysaccharide groups were higher than those in the non-selenium-modified BRP group. This observed enhancement in immune organ indices is consistent with the immunopotentiating effects reported by Zhang et al. for similar selenium-modified polysaccharides [48]. The results indicate that compared to the non-selenium-modified BRP group, all three different doses of sBRP promoted the development of immune organs in broiler chickens, with the high-dose sBRP-H group showing a more pronounced effect.

Lymphocyte proliferation is a protective physiological response to exogenous antigens [49]. A high proliferation rate signifies enhanced cellular immune function, as it reflects antigen-driven clonal expansion of T cells and correlates directly with pathogen clearance and cross-protective immunity [50]. The experimental results showed that both BRP and sBRP can promote lymphocyte proliferation, but sBRP is more effective. When the oral dose was 20 mg/kg, the A_570_ values of the sBRP-H group were higher than those of the BRP group at all time points, and the lymphocyte proliferation rate was significantly higher than that of the BRP group at all time points, reaching a maximum of 41.85%. This is highly consistent with the results reported by Liu et al. [35] on the significantly increased lymphocyte proliferation rate of selenium-enriched Atractylodes macrocephala polysaccharides in vivo and by Gao et al. [51] on the significantly increased lymphocyte proliferation rate of selenium-enriched garlic polysaccharides in vitro. Compared with the BRP group, the significantly increased spleen index in the sBRP-H group also provides a better structural foundation for lymphocyte proliferation.

The humoral immune function of animals is primarily mediated by antibodies produced by B cells, with antibody titers showing a negative correlation with incidence rates (higher titers correlate with lower incidence rates) [52]. The ND vaccine induces high-titer antibodies to block viral attachment and transmission, thereby eliciting strong and durable humoral immune protection [53,54]. Antibody levels serve as a key indicator for evaluating an animal’s humoral immune function. The results of this experiment showed that the HI antibody titers in all groups exhibited a trend of first increasing and then decreasing, reaching a peak at D_21_. During the experiment, the HI antibody titers in the sBRP-H group were significantly higher than those in the Con, Vac, and unmodified BRP groups (*p* < 0.05). The HI antibody titers in the three different dose groups of sBRP exhibited a dose-dependent effect. Notably, at all time points, even the lowest dose of the sBRP-L group elicited higher HI antibody titers than the unmodified BRP group, mirroring the synergistic potentiation previously observed with selenium-modified polysaccharides in ND vaccines [55]. This enhancement of humoral immunity may be related to IL-6-mediated B cell activation: In this study, serum IL-6 levels were consistently elevated in all sBRP groups at various concentrations, with the sBRP-H group showing significantly higher serum IL-6 levels than the Vac and BRP groups, peaking at 54.032 pg/mL. IL-6 has been shown to promote plasma cell differentiation and antibody secretion, suggesting that selenium modification may synergistically enhance humoral immune responses by regulating the inflammatory cytokine network. To directly confirm that sBRP influences plasma cell differentiation via this pathway, future studies should assess IL-6 production locally within the germinal centers of lymphoid tissues such as the spleen.

Interleukins and interferons are a class of cytokines that play a key regulatory role in the immune system, and they are crucial for the regulation of immune responses. IL-2 is involved in the proliferation of T lymphocytes, the differentiation of B lymphocytes into plasma cells, and the promotion of the proliferation of antigen-specific T lymphocytes [56]. IFN-γ is primarily produced by activated T cells and NK cells, and its main physiological function is immune regulation [57]. In this experiment, except for the IFN-γ levels at D_14_, the IL-2 and IFN-γ levels in the high-dose sBRP-H group were significantly higher than those in the Con and Vac groups (Figure 6 and Figure 8). The synergistic increase in IL-2 and IFN-γ indicates that sBRP, as an immunoenhancer, can effectively activate a strong and sustained cellular immune response. IL-6 is produced by activated macrophages, dendritic cells, and other innate immune cells, primarily participating in inflammatory responses and promoting immunoglobulin production. In this experiment, IL-6 levels in the sBRP-H group were significantly elevated compared to the Con, Vac, and BRP groups, reflecting systemic inflammation and innate immune activation in chickens following sBRP administration, thereby creating conditions for adaptive immune responses.

The intestinal epithelial barrier function arises from the collective activities of heterogeneous cell populations: barrier-forming cells (columnar epithelial cells), secretory cells (GCs, Paneth cells, enteroendocrine cells), and immune-related cells (IELs, microfold cells, tuft cells) [58]. Their interactive network underpins gastrointestinal homeostasis. Goblet cells, primarily located in the intestinal mucosal epithelium, are central to secreting mucins (e.g., MUC2) that form the mucus layer covering the epithelial surface [59]. This layer constitutes the primary physical defense of the intestinal mucosal barrier. Intraepithelial lymphocytes (IELs), distributed among intestinal epithelial cells, serve as the first line of mucosal immune defense by recognizing aberrant cells (e.g., infected or cancerous cells) via T-cell receptors and directly eliminating pathogens [60]. This study found that compared to the Con and Vac groups, the sBRP-M group exhibited significantly increased densities of both IELs and GCs. The elevation in both cell types suggests that sBRP acts as a potential oral immune stimulator and enhancer of the Newcastle disease vaccine, which concurrently enhances the physical and immune barrier functions of the intestine. This synergistic mechanism may ultimately boost mucosal vaccine efficacy. Notably, for orally administered vaccines, immunogenicity is often constrained by the physical barrier of the intestine (e.g., the mucus layer) and immune tolerance mechanisms [61]. Goblet cell-associated antigen passages (GAPs) can transport antigens from the intestinal lumen to the lamina propria, thereby initiating systemic immune responses and overcoming these limitations [62]. GAPs play a dual role in intestinal immunity—breaching the barrier and enabling precise immune regulation—providing a crucial target for oral vaccine design. The observed increase in goblet cell density in this study directly strengthens intestinal immune defense capacity, consequently enhancing vaccine immunogenicity.

The findings indicate that the oral administration of sBRP significantly enhances the immunogenicity of the Newcastle disease vaccine. The observed robust cell-mediated immunity (elevated IFN-γ and IL-2) suggests that oral co-administration successfully leverages the gut-associated lymphoid tissue (GALT) to initiate a protective immune cascade [63,64], which is critical for combating mucosally encountered pathogens like NDV. The concurrent boost in mucosal defenses—illustrated by the increased densities of intraepithelial lymphocytes and goblet cells—further demonstrates the unique advantage of the oral route in establishing frontline immunity at the primary site of pathogen entry [65]. Although the specific effects on Th2 immunity merit further investigation, the observed potent humoral response, reflected in elevated HI titers, confirms the ability of sBRP to promote broad-based immune enhancement. The ability to provoke a mixed immune response indicates that sBRP may have applications not only for viral vaccines but also for bacterial vaccines, especially those necessitating a robust Th1-polarized response for effective clearance [66,67]. Additionally, the promising efficacy of this oral regimen invites further investigation into alternative methods of administration, such as parenteral routes, which may lead to an even more pronounced systemic antibody response. Future research should aim to clarify the exact mechanisms involved, refine delivery methods, and assess effectiveness across various vaccine platforms, including those targeting bacteria.

## 5. Conclusions

This study successfully prepared sBRP using the HNO_3_-NaSeO_3_ method, with the highest selenium content reaching 30.6 mg/g. A series of structural characterizations indicate that selenium is covalently modified to the polysaccharide backbone in the form of Se-O-C. In vivo studies demonstrated that, compared to other groups, the sBRP group enhanced the immune organ indices, cellular immunity (lymphocyte proliferation capacity), humoral immunity (HI antibody titers and serum levels of IL-2, IL-6, and IFN-γ), and mucosal immunity (IELs and goblet cell densities) in yellow-feathered broilers, indicating superior immune responses. This study provides a theoretical basis for the development and application of novel selenium-enriched polysaccharide immunopotentiators. Future work should explore alternative delivery methods for sBRP and its potential as a broad-spectrum immunoenhancer for viral and bacterial vaccines.

## Figures and Tables

**Table 1:** Grouping of experimental animals and administration procedure.

Group	Medicines (on Days 14 and 28; Oral Gavage)	Dose
Con	normal saline	0.5 mL
Vac	vaccine + normal saline	0.5 mL
BRP	vaccine + BRP	0.5 mL, 20 mg/kg
sBRP-L	vaccine + sBRP	0.5 mL, 5 mg/kg
sBRP-M	vaccine + sBRP	0.5 mL, 10 mg/kg
sBRP-H	vaccine + sBRP	0.5 mL, 20 mg/kg

Note: Vaccine, Newcastle Disease Vaccine (Strain La Sota) administered via drinking water. The oral gavage administration regimen (for BRP, sBRP, and saline) commenced concurrently with each vaccination (on days 14 and 28) and was performed once daily for five consecutive days.

**Table 2:** The yields, contents of carbohydrate and selenium of nine sBRPs.

sBRPs	A Na_2_SeO_3_ (mL)	B Temp (°C)	C Time (h)	Rate of Yield (%)	Carbohydrates (%)	Se Content (mg/g)
sBRP_1_	4	40	4	36.66	65	28.4
sBRP_2_	4	60	6	14.72	60	6.94
sBRP_3_	4	80	8	24	59.5	21.5
sBRP_4_	6	40	6	36	44	17.9
sBRP_5_	6	60	8	11.26	49.5	30.6
sBRP_6_	6	80	4	28.57	18.98	25.7
sBRP_7_	8	40	8	18	23.75	19.8
sBRP_8_	8	60	4	12.8	15.88	10.2
sBRP_9_	8	80	6	32	45.02	14.9
K1	18.95	22.03	21.43			
K2	24.73	15.91	13.25			
K3	14.97	20.70	23.97			
R	9.77	6.12	10.72			

Note: The L_9_(3^4^) orthogonal design evaluated three factors (A: 0.05 g/mL Na_2_SeO_3_ addition (mL), B: Temperature (°C), C: Time (h)) at three levels each.

**Table 3:** The lymphocyte A_570_ values of every group.

Groups	D_7_	D_14_	D_21_	D_28_
Con	0.10333 ± 0.001 c	0.10067 ± 0.001 ^b^	0.10417 ± 0.001 ^b^	0.10133 ± 0.001 ^d^
Vac	0.10583 ± 0.001 ^c^	0.10467 ± 0.001 ^ac^	0.10933 ± 0.001 ^c^	0.10367 ± 0.001 ^b^
BRP	0.11683 ± 0.003 ^abc^	0.10650 ± 0.001 ^ab^	0.12833 ± 0.002 ^d^	0.10700 ± 0.001 ^ac^
sBRP-L	0.11750 ± 0.002 ^ab^	0.10667 ± 0.002 ^ab^	0.15317 ± 0.017 ^abcd^	0.10633 ± 0.001 ^abc^
sBRP-M	0.12233 ± 0.001 ^ab^	0.11283 ± 0.004 ^ab^	0.15383 ± 0.010 ^ad^	0.10867 ± 0.002 ^abc^
sBRP-H	0.13467 ± 0.013 ^abc^	0.11500 ± 0.003 ^a^	0.15433 ± 0.013 ^acd^	0.11150 ± 0.003 ^ac^

Note: Data are presented as mean ± SEM (*n* = 6). Column data without the same superscripts (a–d) differ significantly (*p* < 0.05, ANOVA). All time points (D_7_, D_14_, D_21_, D_28_) are expressed as days post-initial immunization. The first immunization was administered on day 14 of the broilers’ age.

**Table 4:** The serum antibody titer of every group (Log2).

Groups	D_7_	D_14_	D_21_	D_28_
Con	2.17 ± 0.200 ^c^	2.67 ± 0.334 ^d^	2.34 ± 0.253 ^b^	2.33 ± 0.232 ^d^
Vac	3.17 ± 0.369 ^bc^	3.17 ± 0.477 ^cd^	3.50 ± 0.514 ^b^	3.50 ± 0.246 ^cd^
BRP	3.50 ± 0.514 ^abc^	3.67 ± 0.334 ^bcd^	3.87 ± 0.439 ^b^	3.83 ± 0.338 ^bc^
sBRP-L	4.33 ± 0.506 ^ab^	3.83 ± 0.307 ^abc^	5.53 ± 0.600 ^a^	4.83 ± 0.525 ^abc^
sBRP-M	4.67 ± 0.593 ^ab^	4.50 ± 0.342 ^ab^	5.67 ± 0.738 ^a^	5.17 ± 0.719 ^ab^
sBRP-H	5.00 ± 0.693 ^a^	4.84 ± 0.307 ^a^	6.23 ± 0.738 ^a^	5.50 ± 0.471 ^a^

Data are presented as mean ± SEM (*n* = 6). Column data without the same superscripts (a–d) differ significantly (*p* < 0.05, ANOVA). All time points (D_7_, D_14_, D_21_, D_28_) are expressed as days post-initial immunization. The first immunization was administered on day 14 of the broilers’ age.

## Data Availability

The original contributions of this study are included in the article. Further inquiries can be directed to the corresponding author.
